# Utilization of Indocyanine Green (ICG) Fluorescence in Patients with Pediatric Colorectal Diseases: The Current Applications and Reported Outcomes

**DOI:** 10.3390/children11060665

**Published:** 2024-05-29

**Authors:** Elizaveta Bokova, Ismael Elhalaby, Seth Saylors, Irene Isabel P. Lim, Rebecca M. Rentea

**Affiliations:** 1 Comprehensive Colorectal Center, Department of Surgery, Children’s Mercy Hospital, Kansas City, MO 64108, USAielhalaby@cmh.edu (I.E.);; 2 Tanta University Hospital, Faculty of Medicine, Tanta University, Tanta 31527, Egypt; 3 Department of Surgery, University of Missouri-Kansas City, Kansas City, MO 64108, USA

**Keywords:** anastomosis, anorectal malformation, fluorescence, Hirschsprung, ICG, pediatric surgery, perfusion, vaginal replacement, cloaca, antegrade continence enema

## Abstract

In pediatric colorectal surgery, achieving and visualizing adequate perfusion during complex reconstructive procedures are paramount to ensure postoperative success. However, intraoperative identification of proper perfusion remains a challeng. This review synthesizes findings from the literature spanning from January 2010 to March 2024, sourced from Medline/PubMed, EMBASE, and other databases, to evaluate the role of indocyanine green (ICG) fluorescence imaging in enhancing surgical outcomes. Specifically, it explores the use of ICG in surgeries related to Hirschsprung disease, anorectal malformations, cloacal reconstructions, vaginal agenesis, bladder augmentation, and the construction of antegrade continence channels. Preliminary evidence suggests that ICG fluorescence significantly aids in intraoperative decision-making by improving the visualization of vascular networks and assessing tissue perfusion. Despite the limited number of studies, initial findings indicate that ICG may offer advantages over traditional clinical assessments for intestinal perfusion. Its application has demonstrated a promising safety profile in pediatric patients, underscoring the need for larger, prospective studies to validate these observations, quantify benefits, and further assess its impact on clinical outcomes. The potential of ICG to enhance pediatric colorectal surgery by providing real-time, accurate perfusion data could significantly improve surgical precision and patient recovery.

## 1. Introduction

In pediatric surgery, the meticulous evaluation of tissue perfusion is critical, particularly in complex surgeries involving anastomoses, colonic pedicles, and mobilization/pull-through techniques, with a heightened risk of compromised blood flow contributing to poor surgical outcomes [1]. The challenge is further exacerbated when addressing congenital anomalies, where abnormal anatomical structures complicate the assessment of tissue viability.

Traditionally, surgeons have relied on subjective indicators such as the color of the serosal surface, bowel peristalsis, arterial pulsation, and bleeding to assess tissue vascularization during gastrointestinal procedures [2,3]. Unfortunately, these methods offer limited accuracy; studies have shown that relying solely on visual cues could lead to an 11% accuracy rate in predicting tissue viability, highlighted by the occurrence of postoperative anastomotic leak [3]. While Doppler ultrasound has been employed to overcome these limitations, its utility remains constrained by several factors, making it less favorable for comprehensive intraoperative perfusion assessment.

Indocyanine green (ICG) fluorescence imaging presents a significant advancement in this arena. It has emerged as a valuable tool in pediatric colorectal surgery, offering a more objective and reliable method for visualizing blood supply. This review delves into the applications of ICG utilized during the surgical reconstruction of pediatric colorectal conditions, underscoring its potential to augment intraoperative assessment and improve surgical outcomes.

## 2. Materials and Methods

A narrative review of the literature published from 1 January 2010 to 1 March 2024, in Medline/PubMed, Google Scholar, Cochrane, and EMBASE databases, including original studies, meta-analyses, randomized controlled trials, and systematic reviews, was performed focusing on manuscripts and books published over the last 5–10 years in English. Search keywords included: “indocyanine green”, “anorectal malformation”, “posterior sagittal anorectoplasty”, “Hirschsprung disease”, “pull-through”, “cloaca”, “cloacal malformation”, “bladder augmentation”, “urinary continence channel”, “Mitrofanoff”, “Monti”, “antegrade continence enema”, “Malone”, “appendicostomy”, “Neomalone”, and “neoappendicostomy”. The reference lists of the retrieved articles were checked for other relevant articles not found during the initial search. Manuscripts and book chapters providing novel insights or addressing current practices in the field were prioritized. Eighty of the selected articles and book chapters were included in the current review. The data were reported in a narrative format focusing on ICG utilization in patients with pediatric colorectal conditions.

## 3. Background

Doppler ultrasound, introduced to overcome subjective visual assessments in surgery, has its limitations, such as the need for direct tissue contact and potential misinterpretation from adjacent vessel signals [1]. Significant advances have been made in the use of indocyanine green (ICG) since its first reported use for liver function tests in the 1950s. [2]. ICG is a water-soluble tricarbocyanine molecule that emits fluorescence when excited by near-infrared (NIR) light at around 800 nm. When injected intravenously, it has excellent uptake in the blood due to plasma protein binding with a half-life of 3–4 min, which limits its uptake in other tissues. Intravenous ICG bound to plasma proteins is absorbed by hepatocytes and undergoes rapid hepatic clearance with biliary excretion [3].

After extensive research, ICG Fluorescence Angiography (ICG-FA) has been proven to be safe for adult patients [4] and shows promise in pediatrics, even in infants under three months, with a systematic review supporting its effectiveness and safety (varying weight-based doses up to 0.5 mg/kg with a maximum daily dose of 2 mg/kg) [5].

## 4. ICG in General Pediatric Surgery

ICG fluorescence imaging significantly enhances surgical precision across pediatric surgery, offering detailed visualization of anatomical structures with minimal complications, low costs, and an easy learning curve [6,7,8,9,10,11,12,13,14,15,16,17,18,19,20,21,22].

In biliary surgery, ICG surpasses traditional intraoperative cholangiography by avoiding ductal cannulation and providing clear visualization in inflamed fields, enhancing safety and cost-effectiveness [23].

In laparoscopic gastrointestinal surgery, ICG enables real-time evaluation of bowel anastomoses, potentially reducing anastomotic leaks [24,25,26], and assists in accurately identifying critical structures like the biliary and urinary tracts, reducing the risk of iatrogenic injury [27].

Studies highlight ICG’s role in liver transplantation, indicating its ability to detect vascular issues not visible on ultrasound and its association with lower risk of primary graft dysfunction [28]. Additionally, systematic reviews and retrospective studies showcase ICG’s effectiveness in reducing anastomotic leaks in colorectal surgery [29] and improving thoracoscopic pulmonary segmentectomy outcomes by better defining resection margins and aiding in the resection of non-palpable pulmonary nodules [30,31]. In pediatric urology, ICG facilitates varicocele repairs, partial nephrectomies, tumor resections, and lymph node biopsies, underscoring its broad utility [20,32].

## 5. ICG in Pediatric Colorectal Surgery

The current and potential applications of ICG technology in pediatric colorectal patients are demonstrated in Table 1.

### 5.1. Hirschsprung Disease

In Hirschsprung disease (HD) treatments, ensuring a well-vascularized connection between bowel segments, is pivotal for successful anastomosis and minimizing complications like leakage or stricture, affecting up to 19% of cases [46,47].

**Figure 1:**
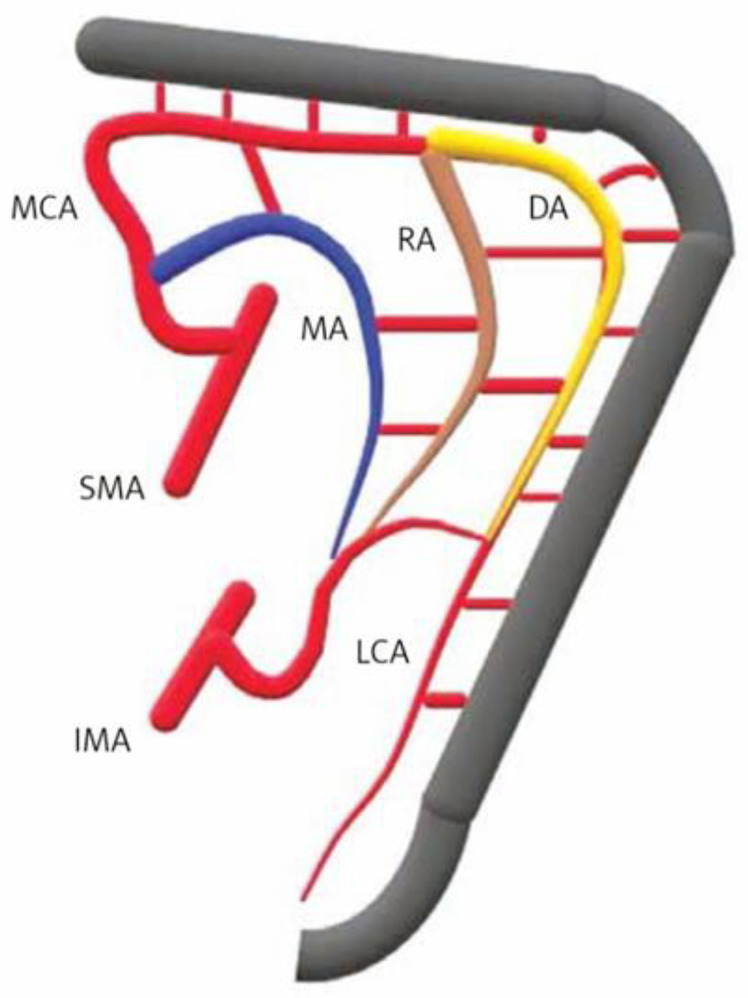
Collateral vessels at the splenic flexure: Drummond artery (DA), Riolan’s arch (RA), and Moskowitz artery (MA) ensuring connection between the superior (SMA) and inferior (IMA) mesenteric artery watersheds. LCA—left colic artery; MCA—middle colic artery. Reprinted from [51].

**Figure 2:**
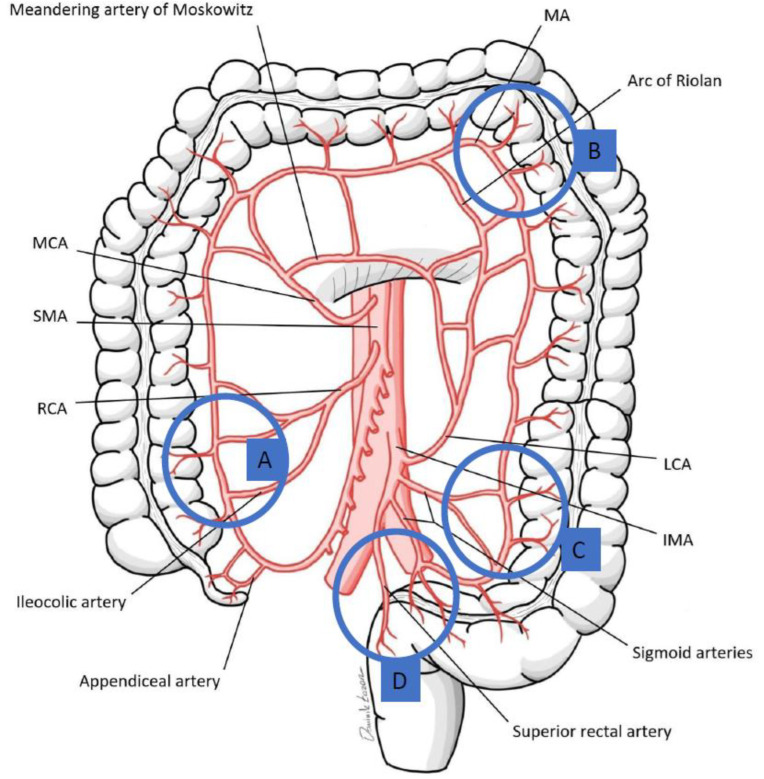
Variability of the marginal artery (MA): (**A**) MA inconsistently present in right colon (**B**) MA can be absent at Driffith’s point (watershed area between SMA and IMA branches) (**C**) MA can be absent at Driffith’s point (watershed between the SMA and IMA branches); and (**D**) MA can be absent at Sudeck’s point (watershed area between the last sigmoid artery and superior rectal artery. Modified from [50].

Determining the resection level in colorectal surgery hinges on evaluating the colonic blood supply, primarily through the marginal artery that links the superior and inferior mesenteric arteries (SMA and IMA) [48,49,50]. The arterial collateral pathways of Drummond’s artery, Riolan’s arch, and the Moskowitz artery are key to maintaining colonic perfusion and providing connections between SMA and IMA territories [50,51,52,53] (Figure 1). The variability in the anatomy of the marginal artery necessitates detailed preoperative assessment to ensure successful anastomosis and optimal surgical outcomes. This is especially true in areas prone to perfusion challenges, such as the rectosigmoid junction, sigmoid colon, splenic flexure, and ileocolic region, where the adequacy of arterial connections can significantly influence the viability of anastomoses (Table 2, Figure 2A,B). Specifically, the convergence point at Sudeck’s critical point and Griffith’s point may lack robust arterial connections, posing risks to the splenic flexure’s blood supply [54,55,56,57]. Sudeck’s critical point at the rectosigmoid junction may lack adequate arterial connections in 5% to 38% of patients [54,55], impacting anastomosis viability. Similarly, at Griffith’s point, where the splenic flexure’s blood supply is at risk, the marginal artery may be absent (7–12%) or weak (22–32%) [56,57], potentially compromising perfusion if larger vessels are ligated. Variability also affects the sigmoid and ileocolic regions, with the sigmoid colon’s marginal artery being adequately wide (≥1 mm) in only 69% of cases, and the right colon’s marginal artery present in just 30% of individuals [56,58]. In cases requiring tension-free anastomosis near the splenic flexure, colonic derotation or Deloyer’s maneuver, which includes the resection and counterclockwise rotation of the right colon around the SMA axis, may be applied to ensure perfusion by preserving the ileocolic artery [59,60,61,62,63,64].

To enhance tissue perfusion assessment in Hirschsprung disease (HD) pull-through surgeries, Indocyanine Green Fluorescence Angiography (ICG-FA) has been effectively employed [34,36,39]. In one prospective study, eight patients undergoing primary pull-through surgeries received ICG via bolus injections, dosed at 0.25–2.5 mg/kg according to body weight, with the possibility of up to six additional doses per procedure if initial fluorescence was inadequate [33]. The study highlighted a median time of 32 s (interquartile range 21–45 s) to achieve optimal fluorescence, directly impacting the surgical approach in 7% of cases by enabling the conservation of well-vascularized bowel segments [33]. Furthermore, ICG-FA has been utilized in various pull-through surgeries, including J-pouch, Swenson, and Soave procedures, at dosages between 0.01 and 0.5 mg/kg [35,37,38]. A recent retrospective single-institution study demonstrated that ICG-FA resulted in a change in the surgical plan in half of the cases during redo pull-through surgery in pediatric patients with Hirschsprung disease by guiding the surgeon to resection on average 10 cm proximal to the initially intended resection margin to avoid poorly-perfused tissue [37].

### 5.2. Anorectal Malformations

Infants diagnosed with anorectal malformations (ARMs) are typically candidates for early-life reconstructive surgeries. These surgeries involve the excision of any existing fistulae and repositioning of the rectum to the anal sphincter complex, all while carefully maintaining the intestinal vasculature. In ARM, a unique aspect of blood supply is that the rectum is often vascularized for several centimeters intramurally. The rectum’s blood supply primarily comes from the superior hemorrhoidal artery (a branch of the IMA) and the middle and inferior hemorrhoidal arteries (branches of the internal iliac arteries). Extensive rectal dissection often compromises the middle and inferior hemorrhoidal arteries, making the distal rectum reliant on intramural flow from the superior hemorrhoidal artery, delivered through the left colic and sigmoid vessels.

#### 5.2.1. Anorectoplasty

During anorectoplasty, relocating the bowel to connect with the anal region presents obstacles akin to those in Hirschsprung disease (HD) surgeries, notably concerning the marginal artery’s significance (refer to Section 5.1). Recent findings from three studies have shown that intravenous ICG usage in five patients undergoing posterior sagittal anorectoplasty (PSARP) or laparoscopic-assisted anorectoplasty (LAARP) enhanced the surgical view, with dosages varying from 0.01 to 0.3 mg/kg [37,39] (Table 2).

ICG’s role extends beyond vascular imaging; its application through the distal colostomy or urinary system can significantly improve fistula visualization, crucial for successful reconstruction. The introduction of laparoscopic techniques has led to a higher identification rate of remnants of the original fistula remnants (ROOFs), with 16 out of 180 (9%) ARM patients reporting post-PSARP complications like urinary and fecal incontinence linked to ROOFs. These issues often stem from premature proximal fistula ligation far from the urethra, leaving a remnant of the original fistula connected to the urinary system [65]. One study highlighted the efficacy of ICG (1.25 mg) injected through the distal colonic fistula in four patients for precise fistula localization during LAARP [41].

#### 5.2.2. Colostomy Closure

During the neonatal period, sigmoid colostomy creation involves the disruption of the left colic artery branches, highlighting the importance of a meticulous evaluation of the vascular supply before undertaking colostomy closure. To secure a length sufficient for the bowel segment to be pulled through, it is vital to preserve both the inferior mesenteric artery (IMA) and the marginal artery during colonic dissection. A study reported the effectiveness of intravenous ICG administration at a dose of 0.3 mg/kg for visualizing vascular perfusion, with assessments conducted within 30 s following injection [42].

In children with a history of ARM and previous descending or sigmoid colostomy, there is a notable interruption in the colonic vascular arcade. This division implies that the rectosigmoid’s most distal part receives its entire blood supply from the IMA. Consequently, ligating the IMA vessels to lower the rectosigmoid segment could lead to rectal loss.

During reconstructive surgery, mobilizing a high rectum to provide adequate operative length involves ligating peripheral branches of the IMA close to the rectal wall, while ensuring the preservation of at least one or two proximal branches. This technique depends on the rectum’s intramural blood supply for vascularization. Any damage to the rectal wall during this process could jeopardize the distal intramural blood flow, underscoring the critical nature of preserving vascular integrity throughout the surgery.

### 5.3. Cloaca

Visualization of tissue perfusion is essential in surgical reconstruction of patients with cloaca, given the multiple pedicles and separation from the common channel. A thorough grasp of vascular anatomy and perfusion assessment is key.

In posterior sagittal anorectovaginourethroplasty (PSARVUP) procedures, it is critical to maintain the vascular supply to the vagina, urinary tract, and bowel. The vagina’s vascularization primarily comes from the uterine vessels and round ligaments, which are at risk of damage, especially in cases with a high common channel. The rectum, dependent on intramural blood flow, requires meticulous dissection to avoid ischemia.

A study highlighted the effectiveness of utilizing ICG fluorescence to assess vascular supply during PSARVUP surgeries, both initial and revisional, in nine patients. In three instances (33%), ICG application led to a change in the surgical approach by identifying inadequate blood flow to the distal colon, thereby indicating the need for repositioning the pulled-through segment to a more proximal colonic site [37].

### 5.4. Vaginal Replacement

The vagina can be reconstructed with a rectum, colonic segment, or small bowel, to name a few bowel-based options. Vascular perfusion of these tissues is evaluated by ICG-fluorescence, with doses administered between 0.1 and 0.3 mg/kg to assess perfusion during neovagina creation [37]. In reported instances, ICG application influenced surgical decisions by revealing the potential issues of viability of the initially chosen rectal tissues for vaginal reconstruction in one out of two cases. Additionally, the effectiveness of ICG in ensuring adequate vascularization was demonstrated during a sigmoid colon vaginoplasty procedure in a patient with Mayer–Rokitansky–Kuster–Hauser (MRKH) syndrome, underscoring its role in minimizing perfusion-related complications [44].

Beyond vascular assessment, ICG has been employed to enhance the visualization of the urinary tract, acting as an invaluable diagnostic tool for identifying concurrent anomalies. One study outlined the use of ICG for ureteric mapping, applied through cystoscopic access, which supported anomaly screening and aided in the careful dissection of the retrovesical space. This approach has significantly mitigated the risk of inadvertently harming the urinary tract during surgery [43].

## 6. Potential Applications of ICG for Bowel and Bladder Management

While the utilization of ICG is increasingly prevalent across various areas of pediatric colorectal surgery, numerous procedures could potentially gain advantages from its application. Certain cases in pediatric colorectal surgery are inherently complex and may require surgical intervention for issues such as constipation, fecal incontinence, and urinary incontinence at various stages of childhood and adolescence [66,67], and, therefore, the assessment of bowel perfusion during surgical reconstructions.

### 6.1. Antegrade Continence Enemas

An antegrade continence enema (ACE) procedure involves establishing a connection between the bowel and the skin, facilitating the administration of flushes to empty the colon at specified intervals. This approach aims to achieve social continence in patients who have not responded to medical management with rectal enemas and laxatives. The connection can be established using various techniques, including the use of the appendix (appendicostomy), a cecal flap (neoappendicostomy), or the cecum itself (cecostomy) [68,69,70].

The choice of ACE type depends on several factors, including (1) the presence or absence of the appendix, (2) the condition of the appendix in terms of its length, lumen, and vascular supply, and (3) the necessity of using the appendix for concurrent procedures such as a Mitrofanoff appendicovesicostomy. In multidisciplinary centers, preoperative planning considers the requirements of both colorectal and urology teams. It is not uncommon for patients requiring antegrade access for colonic emptying to have associated urologic anomalies, necessitating both antegrade flushes for bowel management and a Mitrofanoff channel for bladder emptying [71,72].

In children requiring simultaneous creation of a Mitrofanoff and a Malone ACE, the appendix will need to be divided and used for both the urinary and stool channels [73]. Studies, however, note a 47% complication rate with higher frequency of revisions as compared to isolated Mitrofanoff or Monti channels, with stomal stenosis accounting for 65% [74]. Insufficient vascular supply to the flap used in such procedures could contribute to these complications, suggesting that ICG’s use might enhance the understanding of flap vascularity and help reduce postoperative issues.

### 6.2. Urinary Reconstruction

Regarding urinary reconstruction, a significant number of individuals with an ARM face urological and renal challenges, including neurogenic bladder. In cases like cloaca, bladder outcomes hinge not just on the surgical technique or channel length but also on associated spinal abnormalities. Complex surgeries might involve creating a continent urinary channel and performing augmentation cystoplasty to expand a neurogenic bladder, reducing kidney damage risks [75]. Although data on ICG use in augmentation ileo-cystoplasty are scarce, one study shows promising outcomes using ICG in orthotopic ileal neobladder reconstruction [76], opening avenues for future research in pediatric settings.

## 7. Protocol for ICG Use in Pediatric Colorectal Surgery

Indocyanine Green (ICG) is recognized for its safety, with minimal toxicity and rare adverse effects, although allergies to iodinated contrasts and shellfish are notable contraindications due to instances of anaphylaxis [2,5,31,77,78]. Despite the increasing utilization of ICG in pediatric colorectal surgery, standardized protocols regarding dosage, timing of administration, number of doses, and administration route are lacking. The senior author has previously described our institutional protocol and outcomes of ICG utilization in children with colorectal conditions [37]. Based on our practices, we demonstrated an algorithm for ICG use in pediatric colorectal surgery in Table 3. The following sections will discuss the existing protocols described by various institutions.

### 7.1. Dose

ICG dosing in pediatric colorectal procedures varies widely, from 0.01 mg/kg to 0.3 mg/kg, even in similar surgeries, indicating the need for dose standardization [37,39] (Table 1). Determining the optimal dose can be complex, with some trials adjusting doses based on patient weight categories (<25 kg, 25–45 kg, and >45 kg) to avoid issues like excessive fluorescence [20,33]. Future research should aim to refine these dosage guidelines to ensure the reproducibility of positive outcomes.

In a recent report of ICG use during stoma closure, adequate blood perfusion for anastomosis was defined by two criteria: (1) the observation of ICG fluorescence within 30 s post-administration, and (2) the intensity of fluorescence being comparable to that of the control area (e.g., distal colon without mesentery dissection). It is therefore critical to establish guidelines for cases where the fluorescence intensity is too weak to detect and to delineate the criteria of blood perfusion adequacy since most studies primarily rely on visual color changes to confirm “adequate vascularity” [42].

### 7.2. Timing

The appropriate interval between ICG injection and achieving observable fluorescence is critical, especially in intraoperative settings. Studies report times ranging from 15 to 40 s for sufficient fluorescence, with some opting for ICG angiography within 1 min post-injection to optimize visualization [33,35,79]. However, consistent documentation of these timings across studies is lacking.

### 7.3. Number of Doses per Procedure

The number of ICG doses administered during surgery varies, with some patients receiving up to five doses depending on the initial fluorescence achieved and within the maximum dosage limits. Practices range, with some suggesting up to six doses per surgery, guided by patient size and total dose limits [33,36].

### 7.4. Route

While intravenous administration is common for assessing tissue vascularity [27], specific surgical scenarios may benefit from alternative routes. For example, enteral ICG administration through a distal colostomy is utilized for visualizing rectourethral fistulas in PSARP procedures, potentially decreasing complications like ROOF [41,65]. Similarly, ICG injection into the urinary system has been employed to safeguard against injuries during dissection and assist in identifying urinary anomalies in patients with MRKH syndrome [43].

## 8. Conclusions

ICG fluorescence imaging has proven beneficial across a spectrum of pediatric colorectal disorders. It has significantly aided surgeries for conditions such as Hirschsprung disease (HD) during pull-through operations, anorectal malformations (ARM) during PSARP or colostomy closure, and cloacal anomalies in PSARVUP or vaginal replacement procedures. The potential for ICG has an expanding scope in pediatric colorectal surgery, with its potential use in urinary reconstructions and ACE and urinary continence channel creation underscoring its expanding role in pediatric colorectal surgery. The route of ICG administration should be defined according to the target structures. Intravenous injection aids in vascular visualization, while the intraluminal route assists in identifying a fistula or other structural anomalies. The wide range of described dosing and assessment of ICG signal adequacy precludes defining the optimal protocol for each procedure. Further multicenter comparative studies are required to establish a standardized procedure-based protocol for ICG utilization in pediatric colorectal surgery. While pediatric colorectal conditions have different pathophysiologies, the use of ICG in the surgical repair of these conditions is aimed at two primary issues: vascular supply and the identification of vital structures such as the urethra and ureter. Despite differences in pathophysiology, these clinical entities benefit from intraoperative applications of ICG, highlighting its versatility. 

## Figures and Tables

**Table 1:** ICG utilization in pediatric colorectal surgery. ARM—anorectal malformation; HD—Hirschsprung disease; ICG—Indocyanine Green; LAARP—laparoscopic-assisted anorectoplasty; MRKH—Meyer–Rokitansky–Kuster–Hauser; N/S—not stated; PSARP—posterior sagittal anorectoplasty; PSARVUP—posterior sagittal anorectovaginourethroplasty; TCHD—total colonic Hirschsprung disease.

Diagnosis	Procedure	Reference	Study Type	*n*	Route	Dose	Time to Sufficient Fluorescence, (Seconds, s)	Comments
HD	Pull-Through	Le-Nguyen et al. [33]	Prospective single-institution clinical trial	8	IV	Per bolus: 0.25 mg/kg–2.5 mg ^1^	32	If fluorescence was insufficient after the initial bolus, another bolus was injected.
		Menon et al. [34]	Retrospective single-institution study	N/S	IV	N/S	N/S	ICG was used in “some” of the reported 28 patients.
		Nakagawa et al. [35]		10	IV	0.01 mg/kg	60	J-pouch in children with TCHD. ICG-FA combined with Lugol’s iodine staining to visualize the anal canal.
		Shafy et al. [36]		N/S	IV	N/S	N/S	
		Rentea et al. [37]		3	IV	0.1–0.3 mg/kg	N/S	Swenson pull-through.
		Muto et al. [38]	Case report	1	IV	1 mL (0.5 mg/kg)	N/S	Soave pull-through.
		Shirota et al. [39]		1	IV	0.01–0.1 mg/kg	N/S	
ARM	PSARP	Paraboschi et al. [40]	Case report	1	IV	1 mg (0.2 mg/kg)	60 ^2^	
		Rentea et al. [37]	Retrospective single-institution study	1	IV	0.1–0.3 mg/kg	N/S	
	LAARP	Li et al. [41]		4	Enteral ^3^	1.25 mg	-	The timing was not reported as the goal was not to assess blood supply but to identify the rectourethral fistula before its ligation.
		Shirota et al. [39]	Case series	3	IV	0.01–0.1 mg/kg	N/S	
	Colostomy closure	Yada et al. [42]		2	IV	0.3 mg/kg	30	
Cloaca	PSARVUP	Rentea et al. [37]	Retrospective single-institution study	8	IV	0.1–0.3 mg/kg	N/S	
MRKH Syndrome	Vaginal replacement	Fontoura Oliveira et al. [43]	Retrospective single-institution study	4	Intra-ureteral ^2^	25 mg	-	ICG was used to visualize the urinary system to prevent its injury during dissection and screen for associated urologic malformations.
		Saxena et al. [44]	Case report	1	IV	0.2 mg/kg	N/S	Total laparoscopic sigmoid colon vaginoplasty.
Rectal Prolapse	Perineal rectosigmoid-ectomy	Yamamoto et al. [45]	Case report	1	IV	0.2 mg/kg	N/S	
Constipation/Fecal Incontinence	Antegrade continence enema procedure * (Malone/Neomalone)							
Urinary Incontinence	Urinary continence channel creation * (Mitrofanoff/Monti)							
	Bladder augmentation *							

^1^ Based on the patient’s weight, a maximum of six doses per procedure. ^2^ The tissue should have been able to maintain the ICG signal for 120 s. ^3^ Via the distal colostomy. * Potential applications in pediatric patients not described in the literature yet.

**Table 2:** ICG utilization in pediatric colorectal surgery. ARM—anorectal malformation; HD—Hirschsprung disease.

Site	Watershed	
Rectosigmoid junction (Sudeck’s point)	Last sigmoid artery (IMA)	Superior rectal artery (IMA)
Sigmoid colon	Sigmoid arteries (IMA)	
Splenic flexure (Griffith’s point)	Middle colic artery (SMA)	Left colic artery (IMA)
Ileocolic region	Ileocolic artery (SMA)	Right colic artery (SMA)

**Table 3:** An algorithm for ICG use in pediatric colorectal surgery based on our institutional protocol and current practices.

Indications:	Evaluate bowel and vaginal tissue perfusion in pediatric colorectal surgeries.
Type of Procedure:	Colorectal resections, anastomoses, and assessment of tissue perfusion.
Administration Route:	Intravenous injection.
Dosage:	0.1–0.3 mg/kg
Advantages:	Provides real-time visualization of tissue perfusion. Helps in assessing the viability of bowel and other tissues, potentially reducing postoperative complications.
Disadvantages:	Variability in imaging intensity depending on the device used. Requires specialized equipment (e.g., Stryker SPY™ system).
Limitations and Precautions:	Device-dependent variations in image quality. Possible allergic reactions to ICG, although rare. Interpretation of fluorescence can be subjective and operator-dependent. No quantitative data available.
Comments:	Bowel (colon/small bowel) and vaginal tissue perfusion were clinically assessed firsthand and then measured using the Stryker system. The tissue perfusion is visualized within 1 to 2 min of intravenous injection [37].

## Data Availability

No new data were created or analyzed in this study. Data sharing is not applicable to this article.
